# Oral mucosal lesions and risk factors in elderly dental patients

**DOI:** 10.15171/joddd.2019.004

**Published:** 2019-04-24

**Authors:** Esin Bozdemir, Hasan Hüseyin Yilmaz, Hikmet Orhan

**Affiliations:** ^1^Department of Dentomaxillofacial Radiology, Faculty of Dentistry, Suleyman Demirel University, Isparta, Turkey; ^2^Freelance Dentist, Bursa, Turkey; ^3^Department of Biostatistics and Medical Informatics, Faculty of Medicine, Suleyman Demirel University, Isparta, Turkey

**Keywords:** Chronic disease, dentures, elderly, oral mucosa

## Abstract

***Background***. The aim of this study was to determine the prevalence of oral lesions in terms of sex, age, educational status, medication use, systemic diseases, the duration of denture use and tobacco or alcohol use.

***Methods***. A total of 709 voluntary patients (375 males and 334 females), aged ≥60 years, were interviewed by one investigator for demographic data, systemic diseases, tobacco or alcohol use, denture use and the duration of denture use.

***Results***. The majority of the participants (87.6%) had one or more oral mucosal lesions. The prevalence of oral mucosal lesions was 46.3% in males and 41.3% in females (P=0.76). The most common oral mucosal lesion was a sublingual varicosity in both males and females. A statistically significant difference was observed between the three age groups (60–64, 65–69, and ≥70 years) with regard to the prevalence of oral mucosal lesions (P=0.02). There was a significant relationship between the presence of systemic diseases and oral mucosal lesions (P=0.01). There was also a significant relationship between denture use and oral mucosal lesions (P=0.001). Smoking and a history of smoking were also significant predictive factors for oral mucosal lesions (OR: 3.385, P=0.045).

***Conclusion***. Although the majority of oral mucosal lesions detected in the present study were benign, there were some patients with premalignant and malignant lesions. Therefore, periodic oral examinations for detection of precancerous and cancerous lesions are important, especially in the elderly, smokers and denture users.

## Introduction

A relationship has been reported between oral mucosal lesions and aging.^1^ Due to reduced immunologic reactivity, impaired DNA repair capacity, impaired carcinogen metabolism and atrophy of oral tissues, particularly of the oral epithelium and the salivary glands, oral mucosal conditions tend to develop more frequently and more rapidly in aging populations.^2^

Age has an important influence on the prevalence of oral mucosal lesions. The prevalence of oral mucosal lesions has been shown to be higher in older subjects than in younger individuals.^1-6^ However, age alone is not the only factor and other findings such as trauma, medications and oral and denture hygiene might affect the development of oral mucosal lesions. Oral mucosal lesions usually occur due to systemic diseases, nutritional disorders, medication side effects or wearing ill-fitting dentures in the elderly.^7^

In addition to age, factors such as gender, educational level, smoking and systemic diseases might pave the way for oral lesions. Since the level of education is an effective element in oral hygiene maintenance, it might be important in the formation of oral lesions. Oral health status is important for the quality of life, owing to physical, social and psychological factors. Early detection and prevention of oral lesions by dental practitioners can improve the quality of life of this population and aid in the attainment of quality aging. The prevalence of oral mucosal conditions is an important parameter in evaluating the oral health of the elderly. Therefore, the aim of this study was to determine the prevalence of oral lesions in relation to sex, age, medication use, systemic diseases, the duration of denture usage, education level, oral hygiene status and tobacco and alcohol use.

## Methods

The study protocol was approved by the Ethics Committee, School of Medicine, the University of Suleyman Demirel. Written informed consent was obtained from all the participants. The subjects were 709 volunteer patients aged ≥60 years and were admitted to the Department of Oral Diagnosis and Maxillofacial Radiology in Suleyman Demirel University Faculty of Dentistry with various dental complaints from March 2008 to April 2009. One oral medicine specialist interviewed all the participants for demographic data, systemic diseases, tobacco or alcohol use, use of dentures and the duration of denture use.

Intraoral examinations of the patients were performed by one investigator. As a part of the routine oral examinations, dental and medical anamneses were taken from the patients. Tongue depressors and mirrors were used to visualize the oral mucosa with palpation being performed where indicated. Radiological examinations were also performed. In the presence of oral mucosal lesions, the diagnosis was made on the basis of the dental/medical history and clinical features according to the World Health Organization Guidelines and Oral Pathology: Clinical Pathologic Correlations.^8,9^ As for smoking, the participants were classified into three categories according to their smoking habits as non-smokers, former smokers and smokers. Oral hygiene was evaluated according to the Oral Hygiene Index (OHI) (Greene and Vermillion) and the DMFT index. The level of education of the participants was classified into six categories as illiterate, literate, primary school, middle school, high school and university. The classification of denture types was performed according to Jainkittivong et al.^7^ Denture wearers were divided into complete denture wearer (CDW) and partial denture wearer (PDW) groups. The CDW group was further divided into subgroups, including maxillary and mandibular complete dentures, and a maxillary or mandibular complete denture alone or in combination with crowns and/or bridges. The PDW group included patients who had either a partial denture alone or in combination with crowns and/or bridges. The duration of denture use was classified into four groups according to Jorge et al^10^ as follows: <5, 6–10, 11–20, and ≥20 years of use. After preparation of a dental treatment plan, the patients were referred to other dental departments according to their requirements.^10^

SPSS 17.0 for Windows (SPSS Inc., Chicago, USA) was used to analyze data. Chi-squared test was used to test for differences. A logistic regression model was used to establish the strength of their effects on the development of oral mucosal lesions. Odds ratios (ORs) were calculated with 95% confidence intervals. Statistical significance was defined as P<0.05.

## Results

The study sample of 709 elderly patients included 375 (53%) men and 334 (47%) women. The patients were divided into three age groups: 60–64 (36.4%), 65–69 (31.6%), and ≥70 years of age (32%).

The majority of participants (44.1%) were primary school graduates. The education level did not significantly affect the presence of oral mucosal lesions (P=0.25).

In terms of bad habits, while 57.1% of the participants never used cigarettes, 33.7% and 9.2% were former smokers and smokers, respectively. The majority of smokers (84.6%) were male and 96.9% of smokers had smoked for more than 10 years. Among the patients, 2.1% declared having an alcohol habit and were defined as drinkers.

With regard to dentures, 76% of the study population wore dentures. In terms of sex, the percentage of males using prostheses (53.2%) was higher than females (46.8%).

The percentage of participants with maxillary and mandibular complete dentures was 39.1% and 28.6%, respectively. Maxillary and mandibular partial denture wearers comprised 28.8% and 35.4% of the study subjects, respectively.

The majority of the subjects with crowns and/or bridges (38.5%) and removable dentures (33.3%) had been using them for ≤5 years. The majority of the elderly with removable dentures (38.8%) were illiterate. The duration of denture use was significantly different in terms of the educational status (P=0.00).

While 78.8% of the elderly population had natural teeth, 21.2% were edentulous. In terms of sex, while the percentage of females who were edentulous was 26.7%, this figure was 16.2% in males. However, 26.4% of the participants had functional dentition (≥20 teeth). The percentage of men with ≥20 teeth (31.6%) was higher than women (20.4%). A total of 470 (84.4%) patients had poor oral hygiene according to the OHI. The majority of subjects with poor oral hygiene (85.8 %) had oral mucosal lesions. There was no significant relationship between oral hygiene and the presence of oral mucosal lesions (P=0.63).

Approximately 87.6% of the participants had one or more oral mucosal lesions. The prevalence of oral mucosal lesions was 46.3% in males and 41.3% in females, with no significant difference (P=0.76). The prevalence of oral mucosal lesions in the three age groups was as follows: 34.8% in the 60–64-year group, 31.6% in the 65–69-year group, and 33.7% in the ≥70-year group. A significant difference was observed between the three elderly age groups regarding the prevalence of oral mucosal lesions (P=0.02).

Red-blue lesions were observed with the highest frequency (45.3%) both in the study population and in both sexes (35.3% for women, 40.4% for men). The most common type of oral mucosal lesion was a sublingual varicosity in both males and females. The second lesion frequently observed was fissured tongue (25.6 % in females, 35.2% in males). While 10% of the patients with mucosal lesions were smokers, 56% did not smoke and 34% had stopped smoking (P=0.09). The most common lesions were sublingual varicosity (9.9%) and denture stomatitis (2.1%) in smokers.

When the determined malignant lesions included adenocarcinoma, melanoma and squamous cell carcinoma, lichen planus was determined as a premalignant lesion in both males and females.

The incidence of systemic diseases was 90%. Cardiovascular diseases were found in 459 (64.7%) participants and often identified as a systemic disease group. The majority of the elderly with systemic diseases (79.7%) had mucosal lesions. There was a significant relationship between the presence of systemic diseases and oral mucosal lesions (P=0.01). The frequencies of mucosal lesions in systemic disease groups in the elderly are shown in Table 1. The majority of the elderly with systemic diseases (91%) had oral mucosal lesions. The prevalence of oral mucosal lesions was higher in denture wearers (78.1%) than in non-wearers (21.9%) (P=0.001).

**Table 1:** The percentages of mucosal lesions in some systemic diseases identified in the elderly

**Disease/disorders**	**Total** **N (%)**	**With mucosal lesion** **N (%)**	**Without mucosal lesion** **n(%)**	**χ** ^ 2 ^ **/P-value**
**Cardiovascular diseases**	459 (64.7)	402 (56.8)	57 (8.1)	0.99
**Bone/joint disorders**	373 (52.6)	330 (46.5)	43 (6.1)	0.45
**Endocrine disorders**	213 (30)	184 (269	29 (4.1)	0.52
**Respiratory diseases**	99 (14)	86 (12.1)	13 (1.8)	0.81
**Gastrointestinal disorders**	240 (33.9)	216 (30.5)	24 (3.4)	0.16
**Neuropsychiatric disorders**	167 (23.6)	144 (20.3)	23 (3.2)	0.54
**Hematologic disorders**	72 (10.2)	64 (9)	8 (1.1)	0.72
**Skin diseases**	53 (7.5)	49 (6.9)	4 (0.6)	0.26
**Genitourinary disorders**	114 (16.1)	105 (14.8)	9 (1.3)	0.11

Results presented as N (%)

A significant relationship was not observed between the presence of oral mucosal lesions and diabetes mellitus (P=0.97). However, there were mucosal lesions in most of the elderly with diabetes mellitus (65.8%). There was a significant relationship between denture use and oral mucosal lesions (P=0.001). Denture wearers exhibited more oral mucosal lesions (68.4%) compared with the elderly without dentures (19.2%). The prevalence of denture-related lesions among denture wearers was 44.2%. The prevalence of denture stomatitis (43.3%) was significantly higher among denture-related lesions. The complete denture wearer group (50.4%) exhibited more denture-related lesions than the partial denture wearer group (35%). Table 2 shows the prevalence of denture-related lesions in denture wearers.

**Table 2:** The prevalence of denture-related lesions in complete and partial denture wearers

	**Complete denture wearer** **N (%)**	**Partial denture wearer** **N (%)**	**Total** **N (%)**
**Denture-related lesions**	116 (50,4)	57 (35)	173 (44)
**Denture stomatitis**	49 (42,2)	42 (73,7)	91 (52,6)
**Denture hyperplasia**	55 (47,4)	5 (8,8)	60 (34,7)
**Traumatic ulcer**	26 (22,4)	7 (12,3)	33 (19,1)
**Angular cheilitis**	10 (8,6)	2 (5,3)	12 (6,9)
**Frictional keratosis**	10 (8,6)	3 (5,3)	13 (7,5)

The subjects who had used their dentures for ≥20 exhibited more denture hyperplasia than other denture wearers. Denture stomatitis was often observed in subjects wearing dentures for 6–10 years. Table 3 shows the distribution of lesions in terms of the age of the dentures. The incidence of all the lesions related to denture use in patients wearing dentures at night (35.6%) was higher than that in patients not wearing dentures at night (8.4%).

**Table 3:** Distribution of denture-related lesions in terms of the age of denture

	**Age of denture**			
	**5 years and over** **N (%)**	**6-10 years** **N (%)**	**11-20 years** **N (%)**	**20 years and over** **N (%)**
**Denture-related lesions**	45 (26)	51 (29,5)	30 (17,3)	47 (27,2)
**Denture stomatitis**	28 (30,8)	30 (58,8)	15 (50)	18 (38,3)
**Denture hyperplasia**	8 (17,8)	13 (25,5)	11(36,7)	28 (59,6)
**Traumatic ulcer**	7 (15,6)	12 (23,5)	6 (20)	8 (17)
**Angular cheilitis**	2 (4,4)	3 (5,9)	1(3,3)	6 (12,8)
**Frictional keratosis**	4 (8,9)	5 (9,8)	1(3,3)	3 (6,4)

The factors associated with an increased risk of mucosal lesions were determined, which included leukoplakia, aphthous ulcers, denture stomatitis, denture hyperplasia and traumatic ulcers. Table 4 shows OR results, statistical significance and confidence intervals of these risk factors. The logistic regression model for having any oral mucosal lesion included the independent variables of duration of denture use, smoking and sex. The risk of having an oral mucosal lesion was higher in individuals wearing dentures for ≥20 years (OR: 4.667, P=0.011) than in subjects wearing them for 11–20 years (OR: 3.045, P=0.036). Smoking and a history of smoking were also significant predictive factors for having an oral mucosal lesion (OR: 3.385, P=0.045). The model for an aphthous ulcer included being a male as a non-significant predictive factor (OR: <1, P=0.617). Wearing dentures for 6–10, 11–20, or ≥20 years was a significant risk factor for denture stomatitis, denture hyperplasia and traumatic ulcers. The model for denture stomatitis and hyperplasia also included diabetes mellitus as a non-significant predictive factor (OR: <1, P=0.338 and OR 1.015, P=0.964, respectively). Medication use was the second predictive risk factor in the model for a traumatic ulcer (OR: 12.787, P=0.012).

**Table 4:** Odds ratios, statistical significance and confidence intervals of risk factors

**Indicator**	**Significance**	**Odds Ratio**	**%95 Confidence intervals**
**Oral mucosal lesion (any)**			
Length of denture use (6‒10 years)	0,258	1,441	0,766‒2,711
Length of denture use (11‒20 years)	0,036*	3,045	1,075‒8,628
Length of denture use (≥20 years)	0,011*	4,667	1,431‒15,218
Non-smokers	0,113		
Former smokers	0,045*	3,385	1,028‒11,149
Smokers	0,394	1,234	0,761‒2,002
Male Gender	0,761	<1	0,596‒1,460
**Aphthous ulcer**			
Male gender	0,617	<1	0,251‒2,270
**Denture stomatitis **			
Length of denture use (6‒10 years)	0,000*	5,158	2,932‒9,075
Length of denture use (11‒20 years)	0,000*	3,960	1,994‒7,868
Length of denture use (≥20 years)	0,000*	4,293	2,242‒8,219
Diabetes Mellitus	0,338	<1	0,427‒1,340
**Denture hyperplasia**			
Length of denture use (6‒10 years)	0,000*	6,847	2,766‒16,952
Length of denture use (11‒20 years)	0,000*	9,946	3,847‒25,714
Length of denture use (≥20 years)	0,000*	29,212	12,652‒67,443
Diabetes Mellitus	0,964	1,015	0,534‒1,929
**Traumatic ulcer**			
Length of denture use (6‒10 years)	0,000*	7,170	2,755‒18,659
Length of denture use (11‒20 years)	0,002*	5,736	1,869‒17,601
Length of denture use (≥20 years)	0,000*	6,905	2,429‒19,625
Medication use	0,012*	12,787	1,735‒94,231

*Statistical significance (P<0.05)

## Discussion

The incidence of oral mucosal lesions ranged from 12% to 61.4% in the epidemiological studies related to the prevalence of oral mucosal lesions in the elderly in our country and abroad.^11-20^ In the present study, this incidence rate was 87.6%, similar to a study by Jainkittivong et al (83.6%).^7^ This incidence was higher than those reported in a few studies (40.7–45.9%) in our country.^18-20^ These variations between studies might be due to a lack of standard methods such as differences in age ranges, the sizes of the study populations, the criteria used to identify oral lesions and the types of oral mucosal lesions.

Males presented with more oral mucosal lesions than females, consistent with Mumcu et al and Dundar and Ilhan Kal.^19,20^ The prevalence of oral mucosal lesions was 46.3% in males and 41.3% in females in the present study. However, there was no significant difference in terms of sex. The incidence of oral mucosal lesions was higher in the 60‒64-year age group than the other age groups, similar to a study by Dündar and Ilhan Kal.^20^ In terms of age groups, there was a significant difference in the incidence of oral mucosal lesions. There was an increase in the incidence of lesions as the age increased in the lesion groups; this was especially observed with ulcers, tongue lesions, cancer and connective tissue lesions. Commonly observed lesions in the elderly in studies in our country and abroad vary from one study to another.^11-20^ Mumcu et al^19^ reported that the most common oral mucosal lesions were lingual varicosities, excessive melanin pigmentation and fissured tongues in the elderly. Fissured tongues, lingual varicosities and denture stomatitis were the most frequently identified lesions in elderly dental patients in a study by Dundar and Ilhan Kal.^20^ Lingual varicosity, fissured tongue and traumatic ulcers were the most common mucosal changes found in an elderly Thai population.^7^ Fordyce granules, herpes labialis, fissured tongue and denture stomatitis have been identified in the 65‒74-year age group in Germany.^2^ Lingual varicosity, frictional keratosis and denture stomatitis were reported in Hong Kong.^12^ Hairy tongue, angular cheilitis, and lingual varicosity were the most frequently determined oral mucosal lesions in Finland.^14^

Fissured tongue and lingual varicosity are the most common oral mucosal lesions in the elderly and the prevalence of these lesions increases with age.^2,7,15,20^ Age is an important risk factor for these two lesions.^19-21^ In the present study, these lesions were detected as the most common oral mucosal lesions.

Denture use was associated with a high prevalence of oral mucosal lesions. Most oral mucosal lesions in the elderly have been correlated with the use of removable dentures. Denture-associated lesions are the most common type of oral mucosal lesions in the aging population. The findings of oral mucosal lesions observed in denture wearers in this study were similar to those previously reported in elderly subjects.^5,7,12,14,15,20,23^ The prevalence of oral mucosal lesions was higher in denture wearers (78.1%) than in non-wearers (21.9%).

While the prevalence of oral mucosal lesions related to denture use was higher in women in some studies,^21,23-25^ in the present study, the prevalence of denture-related lesions was higher in female subjects. This high frequency of lesion incidence among females might be due to the fact that during or after menopause there is atrophy of the oral mucosa, resulting in decreased protection against the chronic irritation of ill-fitting dentures.^22^

The most common denture-related mucosal lesions observed in previous studies included traumatic ulcer, denture-induced stomatitis and denture hyperplasia.^24,26,27^ These findings are similar to our study. The most commonly observed denture-related lesion was denture stomatitis in this study.

In agreement with several studies,^24,25,28^ in the present study there were more denture-related mucosal lesions in complete denture wearers than in partial denture wearers. In contrast to our findings, there were no significant differences in the prevalence rates of denture-related mucosal lesions between the two denture wearing groups in some studies.^20,27^

The length of denture use has been shown to be positively related to increased denture-related mucosal lesions in previous studies.^26,28-30^ However, denture-related mucosal lesions might occur in relation to poor denture hygiene and the continuous use of dentures throughout day and night.^31^ In this study, the individuals who used their dentures for ≥20 years exhibited more denture hyperplasia than other denture wearers. However, the risk of having an oral mucosal lesion was higher in individuals who had used their dentures for ≥20 years than in those who had used them for 11–20 years. The incidence of all the lesions related to denture use in patients who used dentures at night (35.6%) was higher than in patients who did not use dentures at night (8.4%). The reason behind this condition might be the long-term traumatic effect on the tissues from dentures occurring as the result of the use of dentures at night.

Epidemiological studies have shown that tongue lesions constitute a considerable proportion of oral mucosal lesions, and their prevalence rates vary in different regions of the world.^7,32-34^ In the present study, tongue lesions were observed in 35.1% of the elderly. Fissured tongue was the most frequent tongue lesion, similar to previous studies.^7,19,21,33^

The oral cavity is one of the most appropriate locations for the development of oncological diseases. Oral cancer is an age-related disease and 98% of patients are >40 years old. There is a linear increase in the incidence of oral cancer with aging. Oral cancer is more common in men than women in many countries. Malignant lesions were determined in only men in the study by Dundar and Ilhan Kal and in only women in a study by Cebeci et al.^20,35^ In contrast to these studies, malignant lesions were determined in both women and men in our study.

The duration of denture use, smoking and male sex were identified as risk factors for oral mucosal lesions in the logistic regression model in this study. The length of denture use has been shown to create a positive effect on the presence of oral mucosal lesions in the literature.^25,28,30^ The length of denture use was also a significant risk factor for having oral mucosal lesions in the current study. When dentures are used for long periods, xerostomia associated with diabetes mellitus increases the traumatic effect of dentures.^36^ Similar to findings reported by Dundar and Ilhan Kal,^20^ but contrary to those reported by Espinoza et al,^37^ diabetes mellitus and the length of denture use were determined as significant risk factors for having denture stomatitis and denture hyperplasia in this study.

Although the majority of oral mucosal lesions detected in the present study were benign, there were patients with premalignant and malignant lesions. Therefore, it is important to carry out periodic oral examinations for the detection of precancerous and cancerous lesions, especially in the elderly, smokers and denture users. The findings of this study support other studies that have reported positive relationships between age, sex and denture use and the presence of oral mucosal lesions.

## Conflict of Interests

The authors declare no conflict of interests related to the publication of this work.

## Authors’ Contributions

EB, HHY designed and conducted the study and wrote the manuscript. HO made statistical analysis of the data in the study.

## Acknowledgments

None.

## Funding

None declared.

## Competing interests

There are no conflicts of interest

## Ethics approval

The study protocol was approved by the Ethics Committee, School of Medicine, the University of Suleyman Demirel. Written informed consent was obtained from all of the participants.
